# Pharmacovigilance Data From Digital Health Systems: Regulations, Implications, and Opportunities—A TransCelerate Perspective

**DOI:** 10.1007/s43441-025-00907-7

**Published:** 2026-05-04

**Authors:** James Whitehead, Rajesh Ghosh, Anna Monaco, Jackie Grissinger, Inessa Neyman, Tom Umrath, Clint Craun

**Affiliations:** 1 AstraZeneca, Newmarket, England, UK; 2 Genentech/Hoffman LaRoche Ltd., South San Francisco, CA USA; 3 Merck & Co., Inc., Rahway, USA; 4 Johnson & Johnson, New Brunswick, NJ USA; 5 Pfizer Global Pharmaceuticals, New York, USA; 6 Novartis Pharmaceuticals Corporation, East Hanover, NJ USA; 7 TransCelerate BioPharma Inc., West Conshohocken, PA USA

**Keywords:** Digital Health Technologies, Pharmacovigilance, Regulatory landscape, Adverse event reporting, Post-approval safety, Patient safety

## Abstract

Digital health technologies (DHTs) represent a clear potential to advance the science of pharmacovigilance and promote patient safety. A project was created to assess the current landscape for use of DHTs and identify opportunities to use DHTs in fulfilling these objectives. TransCelerate Biopharma Inc, a non-profit launched in 2012 to accelerate and enhance research of human medicines, led an assessment of digital health in pharmacovigilance focused on the global regulatory landscape. This was achieved through first identifying and forming a glossary of applicable terminology and compiling an index of related regulations, guidelines, and source materials from global health authorities. Using this information, a regulatory landscape assessment was performed, and an industry survey designed. Both outputs indicated that the use of DHTs in pharmacovigilance applications in the pharmaceutical industry is in early development stages for both regulations and real-world utilization. This insight led TransCelerate to develop a tool that would enable a robust exploration of individual DHTs used in the post approval setting through the lens of pharmacovigilance professionals, through proposed considerations for enhancing product safety. This work is intended to positively support the industry in understanding the use and impact of DHTs on pharmacovigilance and readiness. There remains an opportunity to set forth guiding principles and a framework, in collaboration with regulators, healthcare professionals, systems, and patients for DHTs to advance the science of pharmacovigilance.

**Supplementary Information:**

The online version contains supplementary material available at 10.1007/s43441-025-00907-7.

## Introduction

Digital health is an innovation expected to improve patient health and outcomes [1]. As with any innovation in healthcare, it creates new benefits and challenges through its implementation across the healthcare spectrum [2]. According to the U.S. Food & Drug Administration, DHTs are defined as any system that uses computing platforms, connectivity, software, and/or sensors for healthcare and related uses. These technologies span a wide range of applications in general wellness in addition to those specifically linked to a medical device. They can be used independently as medical products, as components of medical products, or as an adjunct to other medical products (devices, drugs, and biologics) [3]. While this is the FDA’s definition, health authorities in other countries and regions may have their own standards for classifying DHTs.

DHT use varies widely in the current environment. Some examples include digitizing patient education resources for delivery via a web portal, using a mobile app to track efficacy and safety, and wearing a sensor to monitor cardiovascular activity. Though adoption of DHTs is relatively new, the demand for these technologies, their availability, and their use are expected to grow. As technology becomes more ubiquitous in the healthcare landscape, pharmaceutical companies will continue to innovate and develop ways to benefit patients through DHTs, despite their complexities and challenges. Pundziene et al. analyzed the benefits of DHTs. One finding was that mobile communication is essential for connecting patients with their healthcare providers. However, this is offset by DHTs’ capacity to create massive data sets that require labor-intensive medical review and handling. Furthermore, and as described by Pundziene, the use of DHTs in healthcare is intricate and nuanced as there are data privacy concerns combined with heavy regulation, high liability, and the cultural consideration of individual countries [2]. Taken together, these factors make the use of DHTs challenging. For pharmacovigilance specifically, a critical implication of DHT use is ambiguity over what constitutes an adverse event within the context of data obtained via DHT. This has created potential challenges across the industry over how and what safety information must be reported to regulators. If the DHT is considered a potential innovation, then the Kassekert et al. [4] view on the need for regulatory harmonization and consultation to support artificial intelligence also holds true here.

The content in this Paper is provided for informational purposes only and should not be construed as conveying legal advice. Any party using these materials to determine the regulatory landscape across jurisdictions for purposes of drug development, drug approval, patient safety or any other purposes bears sole and complete responsibility for determining what laws, regulations, and guidances apply to its conduct and operations in each relevant jurisdiction and complying with (including how best to comply with) all applicable laws and regulations in all relevant jurisdictions. The views and opinions expressed herein are those of the authors; they do not necessarily reflect those of their affiliated companies.

## Methodology

**Fig. 1:**
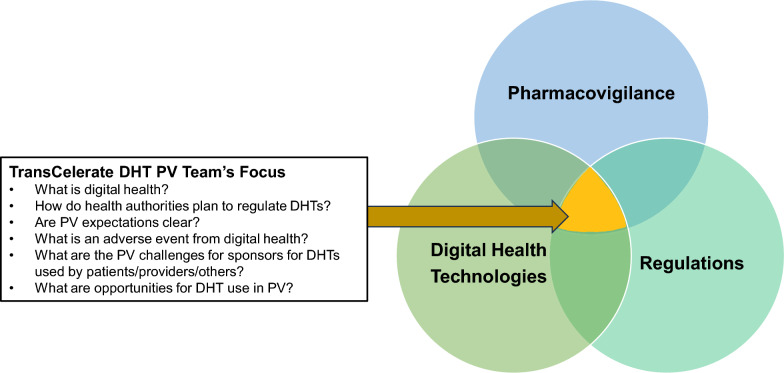
TransCelerate Digital Health Technologies (DHT) Pharmacovigilance (PV) Team’s Focus

In November 2022, the “TransCelerate Pharmacovigilance Data from Digital Health Systems Topic Team” was formed to identify the opportunities and challenges of DHT use in pharmacovigilance across the pharma industry, with the ultimate goal of generating considerations and solutions for voluntary use. The initiative was therefore designed around deliverable-driven workstreams. Figure 1 sets out the project components and objectives [5].

The project’s first step was to catalogue commonly used vocabulary (e.g., digital health, adverse event) in a Glossary with associated synonyms and regulations (Reference Resource Library) related to digital health in general in order to increase clarity, communication, and better decision-making in this space [6, 7]. This established a basis for assessing the global regulatory landscape.

**Fig. 2:**
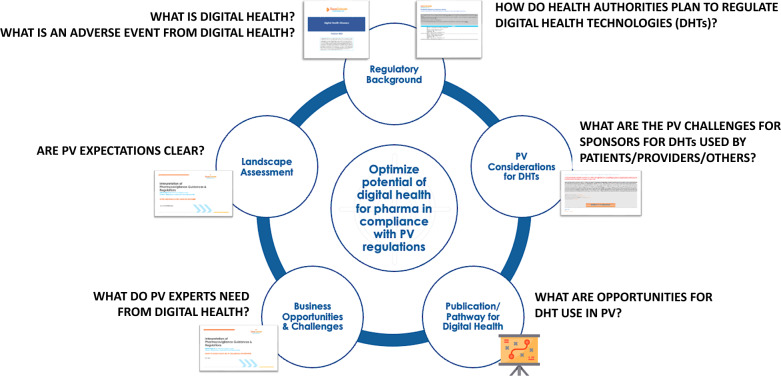
Driving questions

The Landscape Assessment began with a working, but unproven, premise that global regulations and health authority guidance for DHT regarding pharmacovigilance were lacking. The goal was to either validate or refute that hypothesis by evaluating the regulatory landscape from key regional and national regulators across six continents [8]. In parallel with these activities, TransCelerate surveyed 17 member companies concerning their experience with DHTs and the challenges they faced in understanding the application of regulatory requirements and regulator expectations for pharmacovigilance (Survey) [9]. The results were intended to help identify possible solutions to better enable efficient, effective, and high-quality practices that benefit industry stakeholders and ensure patient safety. Several potential tools were identified as possibly beneficial to pharmaceutical companies, with one selected for further development—a Pharmacovigilance Considerations Guide for DHTs [10]. For each step, the TransCelerate team had a set of driving questions to answer, which are presented in Fig. 2.

## Results

### Collection of Digital Health Definitions

**Fig. 3:**
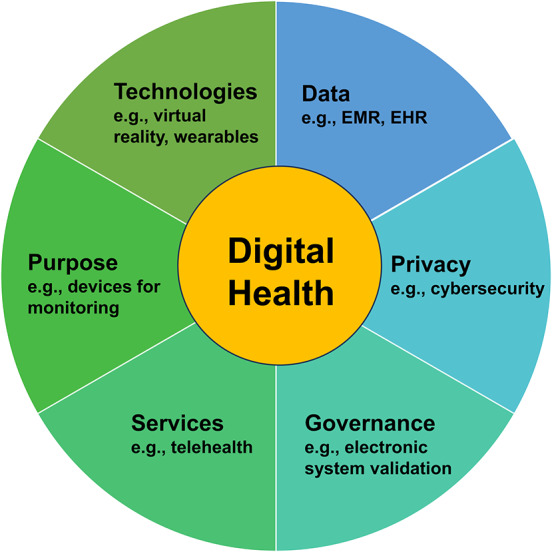
Glossary overview

The final output of this exercise included key terms with definitions (Fig. 3). The findings served to confirm what was found to be a distinct lack of harmonization of DHT terminology across the regulatory environment and in the published literature.

### Regulatory Resource Library and Landscape Assessment of Digital Health in Pharmacovigilance

**Fig. 4:**
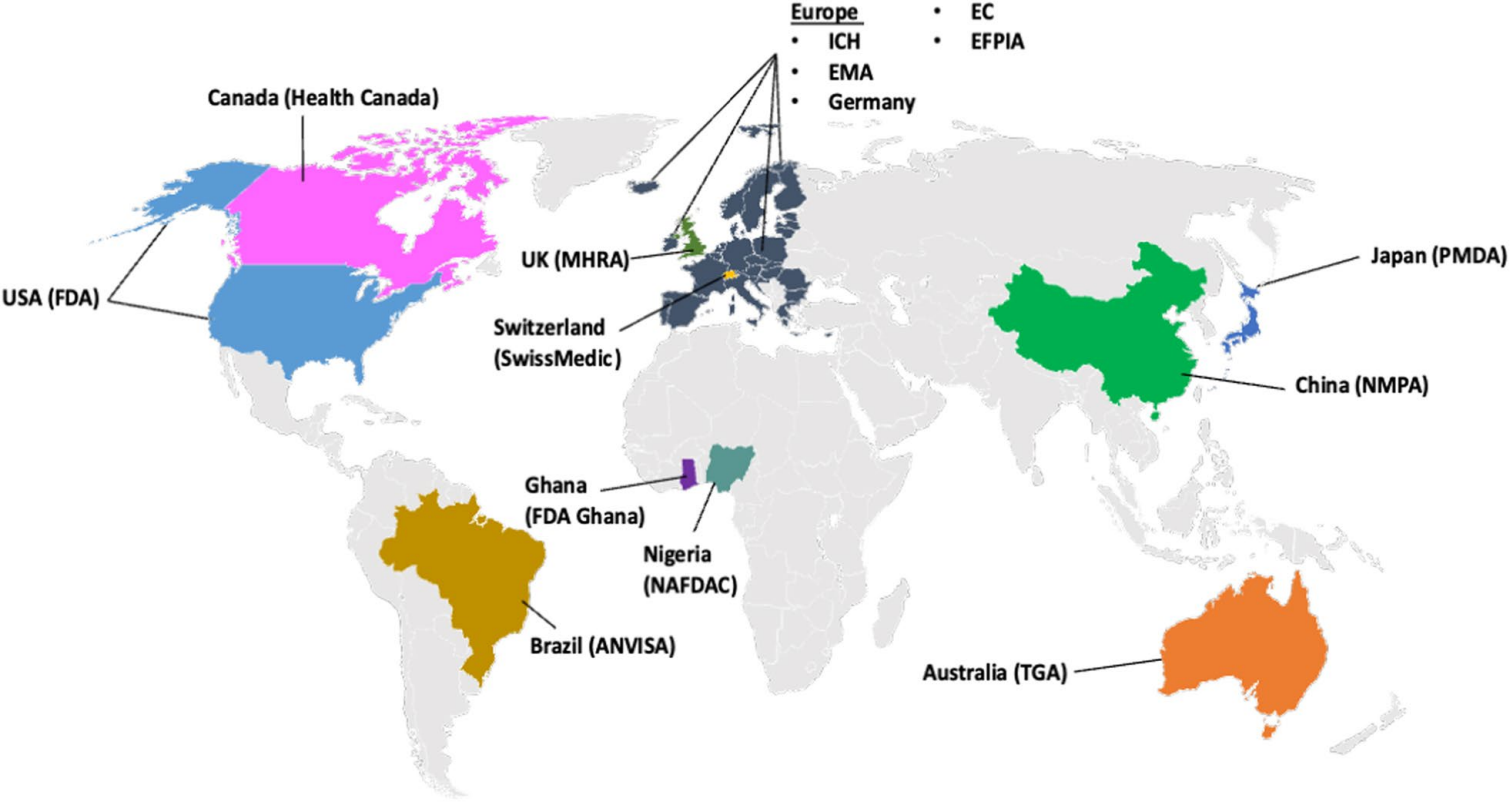
Countries and regions considered in the assessment

**Fig. 5:**
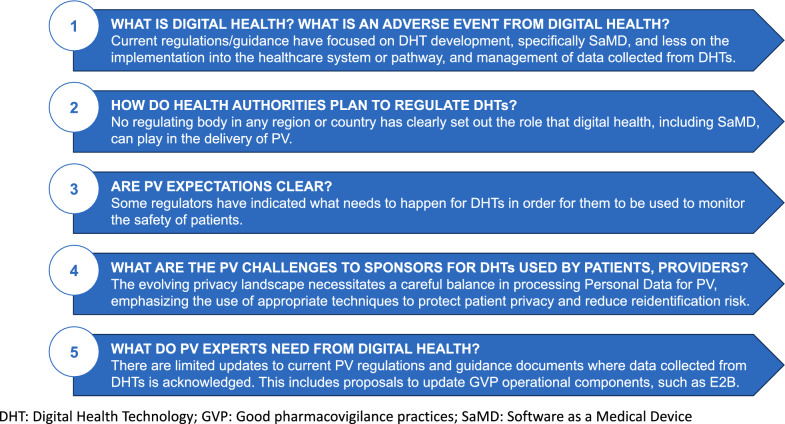
Landscape Assessment key takeaways

During compilation of the regulatory Reference Resource Library, the lack of information specific to pharmacovigilance and DHT supported a preliminary belief that health authority guidance on these topics was limited. The results underscored that improvements could be made to the global regulatory structure to improve uniformity, consistency, and compliance, with an objective of leveraging post-marketing data to further promote the safe use of medical products by patients. The need for alignment is also summarized by Gelis et al. [11] as “a harmonization of the partly disparate regulatory requirements in the US and the EU accompanied by further development of the regulatory landscape in the EU, could further foster the use of digital tools in drug clinical development”. Though some regulators are releasing documentation relevant to digital health in general, none have set forth clear guidance on using digital health in performing pharmacovigilance. Areas of strength were found in the work of ICH E2D, where digital health platforms were defined, and managing adverse events was addressed in a general context [12]. At a country level, there is an acknowledgement in countries such as Ghana, that the implementation of a DHT should consider the impact on the healthcare pathway and once implemented, monitored to ensure safety and performance is delivered for all stakeholders [13] (See Figs. 4 and 5 for an overview of the countries considered and key takeaways).

The second element of the assessment was a professional literature review, which demonstrated there is a growing trend of integrating DHT into existing patient care practices (e.g., telehealth), offering the potential to reach more patients faster and through more convenient channels [13].

In conclusion, the landscape assessment provided insight on the external environment, but it did not resolve uncertainty about how DHT is regulated from a pharmacovigilance perspective; namely how DHT is defined, how it can be used (e.g., with respect to data privacy), how to ensure data quality, and how reporting adverse events can be streamlined with the use of DHTs.

### Industry Survey on Digital Health Use in Pharmacovigilance

**Fig. 6:**
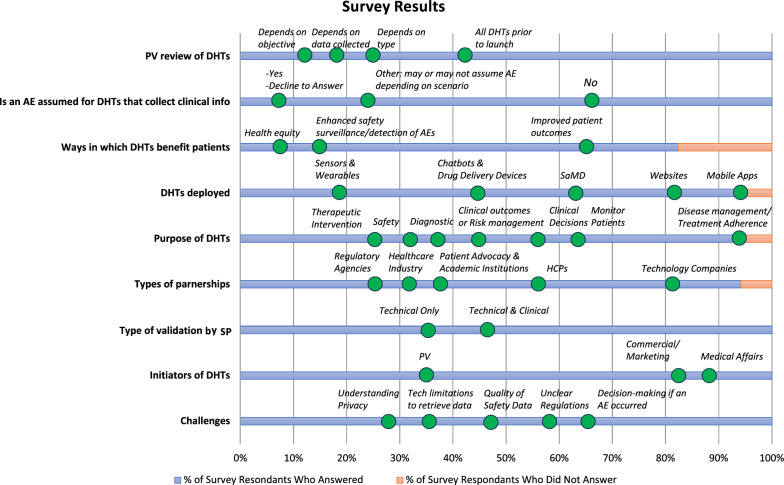
Survey results

A survey completed by 17 companies revealed that while many increasingly use DHTs for a variety of objectives and see the benefit for patients, the industry as a whole is still in the early stages of broadly incorporating DHTs into how pharmacovigilance is delivered or of understanding of their impact on pharmacovigilance compliance [9]. Key findings from the survey are presented in Fig. 6, and are listed below:

1. Companies are primarily deploying DHTs that do not require premarket notification and/or health authority approval, such as websites/applications for disease management or treatment adherence.
2. Most companies indicated that they work with external technology vendors to develop and deploy DHTs.
3. The development process for DHTs includes technical validation; however, clinical validation may not be a part of that development process.
4. Pharmacovigilance departments serve as reviewers for DHTs; however, according to the survey responses, pharmacovigilance does not initiate the development of DHTs in most instances.
5. For DHTs that collect disease symptoms/outcomes/clinical data (e.g., laboratory values, weight gain/loss, heart rate, etc.), most companies do not automatically consider individual deviations from normal ranges to be adverse events without additional context.
6. Companies face challenges with lack of health authority regulations and guidance for determining if certain data qualify as adverse events.

The project team believes this is the first effort to benchmark how DHTs are being used in the post approval setting focused on pharmacovigilance.

### DHT PV Considerations Guide

**Fig. 7:**
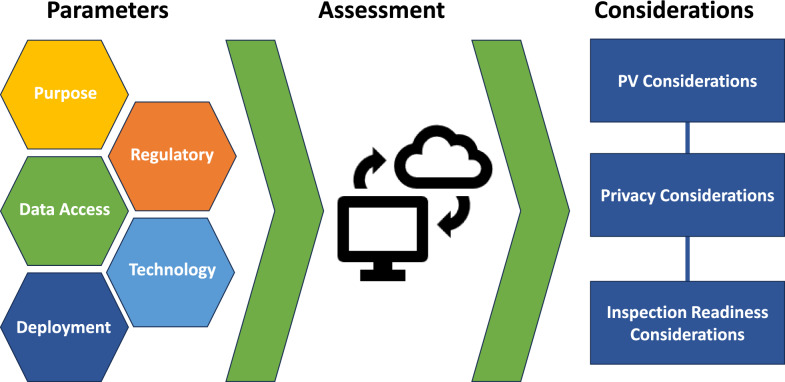
Format of the DHT PV Considerations Guide

The survey showed that while companies increasingly use DHTs for a variety of objectives and see benefits for patients, pharmacovigilance departments have limited involvement in the development of DHTs, acting mainly as reviewers for potential impact to PV systems and practices. Existing regulations and guidance have successfully supported the idea that medicinal products can be applied to DHTs. However, the novelty in their approaches to data collection often pose challenges in interpretation and consistent use by industry. While the potential benefits of DHTs are high, the varied maturity of health authority regulations, specifically those governing digital health in different parts of the world, continues to limit global and widespread harmonization and usage of such tools. The TransCelerate project team has developed a solution for DHT considerations summarized in Fig. 7 [10]. It provides visibility to challenges and opportunities for a range of stakeholders.

### Adverse Events in the Context of DHTs

Based on a summary review of DHT definitions, regulations, and industry practices, there is no clear framework for the identification, collection, and reporting of adverse events in the context of DHTs. A pragmatic way forward is as follows:

- Raw data representing outliers from normal ranges typically would not be considered adverse event information unless the DHT specifically collects adverse event information.
- Sponsors need to set criteria under which adverse events generated by the DHT need to be evaluated and reported.
- In the post approval setting, reporters (HCPs, patients, caregivers) may report any events they consider to be adverse events to pharmaceutical companies.
- DHT safety data should be handled under the company’s existing processes for adverse events, acknowledging DHT as the source of the report.

## Discussion

The project found multiple shared challenges for the pharma industry and other stakeholders related to using DHTs in pharmacovigilance. A series of themes was thereby developed that focus on opportunities for enhancement.

1. *Education*: To realize the value of DHTs in pharmacovigilance, sponsors of medical products must first understand their design, development, and potential implementation. Only once understood and combined with the Good Pharmacovigilance Practice (GVP) expertise can DHTs be fully utilized to advance patient safety.
2. *Shared language*: A common lexicon is needed to support effective discussions between stakeholders. This will avoid misunderstanding and potential non-compliance, especially if the DHT is registered as a medical device with its corresponding regulatory requirements.
3. *Clarity on gaps*: Identifying the gaps in health authority requirements for DHT should help pharmacovigilance departments optimize use of new technologies.
4. *Taking a holistic mindset*: The use of DHTs needs to be considered holistically across a company’s various functions, particularly from a clinical and technical validation perspective. For DHTs to be used effectively, they must be clinically validated. This can be achieved by employing a holistic mindset for design, development, and implementation.
5. *Capitalize on Increasing comfort*: There is increasing comfort within the pharmaceutical and biotechnology sector in exploring the use of DHT. While the field is still evolving, there is corresponding interest and motivation in the expanded use of DHTs. The trend should induce pharmacovigilance departments to play a leading role in DHT design & development.
6. *Designing a digital health safety strategy during clinical development which can be extended once released into the healthcare system*: DHTs for post approval use should be designed, developed, and validated during the clinical development of medical products to support safety and performance assessment. Therefore, pharmacovigilance functions should be engaged early in the process to incorporate a safety strategy that critically analyzes DHT use in diagnosis, monitoring, and treatment. These taction’s have the potential to improve the benefit to risk profile of a medical product.
7. *Collaborating across the care continuum*: With healthcare systems increasingly utilizing DHTs in an evolving regulatory environment, pharmacovigilance departments should develop collaborative channels with all stakeholders involved in patient safety healthcare. This dialogue should begin during the early development of the medical product.
8. *Clarity and approach of adverse event reporting*: DHTs offer opportunities to engage health providers and patients in novel ways that were not available until recently. This has created challenges including definition of adverse events, interpretation of pharmacovigilance regulations across companies, and uncertainty with widespread use. A key objective should be to make the process for adverse event reporting for DHT reflect those received through other sources. This would enable the greatest benefit of DHTs to patients while reducing the burden for industry and regulators.

Finally, there is an opportunity to advance patient safety through the use of DHTs to enhance pharmacovigilance by taking license to engage in partnerships for its optimum utilization. Together with regulators, healthcare providers, professional societies, academia, and patient groups, pharmacovigilance departments should attempt to set collaborative principles and frameworks upon which to operate.

## Conclusion

This initiative focused on assessing the evolving nature of digital health use for pharmacovigilance in the pharmaceutical and biotechnology industries, the regulatory landscape around the globe, and industry practices, in order to identify areas for improvement. The evidence collected through an evaluation of regulations, a literature review, and an industry survey establishes the need for increased clarity and global alignment on regulatory terminology and guidance. Appropriate considerations for governance, audit readiness, privacy, and consultation with relevant experts should be incorporated into deployment plans for digital health tools. The TransCelerate project team has proposed pragmatic approaches for pharma and biotech companies to implement digital health tools in various settings to benefit patients and health providers and to augment clinical development. It is our belief that DHTs can have most positive impact on maintaining and enhancing patient safety if pharmacovigilance departments within pharma companies are consulted early and continuously to evaluate the impact digital health tools have on patient safety and regulatory compliance.

## Supplementary Information

Below is the link to the electronic supplementary material.

Supplementary file1 (PDF 410 KB)

## Data Availability

No datasets were generated or analysed during the current study.
