# Supplementary material for: Pharmacovigilance Data From Digital Health Systems: Regulations, Implications, and Opportunities—A TransCelerate Perspective
Source: Ther Innov Regul Sci. 2026 May 4;60(4):1163–70. doi: 10.1007/s43441-025-00907-7 (PMC13354680; doi:10.1007/s43441-025-00907-7)
Supplement: Supplementary file 1 — Supplementary file1 (PDF 410 KB) [file 43441_2025_907_MOESM1_ESM.pdf]

Electronic Supplemental Material (ESM)

Pharmacovigilance data from digital health systems: Regulations, implications, and opportunities – A TransCelerate perspective

Journal: Therapeutic Innovation & Regulatory Science (TIRS)

Authors

James Whitehead[1], Rajesh Ghosh[2], Anna Monaco[3], Jackie Grissinger[4], Inessa Neyman[5], Tom Umrath[6], Clint Craun[7]

Author Affiliations

[1]AstraZeneca,

[2] Genentech/Hoffman LaRoche Ltd.

[3] Merck & Co., Inc.,

[4] Johnson & Johnson Inc.,

[5] Pfizer Global Pharmaceuticals,

[6] Novartis Pharmaceuticals Corporation,

[7]TransCelerate BioPharma, Inc.

Topic 8: PV Data from Digital Health Systems: Regulations, Implications and Opportunities

Member Company Survey

The purpose of this survey is to collect data from Member Companies about their experience with digital health technologies (DHTs) and challenges faced by Member Companies to understand Pharmacovigilance (PV) requirements/Health Authorities expectations to identify possible solutions to better enable efficient, effective, and high-quality delivery of DHTs that benefit patients. In addition, the data collection may result in publication(s) and/or health authority interactions to bring awareness of Pharmacovigilance challenges with the use of DHTs. To support future publications and/or health authority interactions, some questions will be collecting background information to conceptualize the extent of the use of DHTs and current PV challenges.

Specifically, key objectives of this survey and rationale for the collection of this data:

Objective	Rationale
Quantify the use of DHTs in post marketing setting.	Validate that use of DHTs is occurring in Member Companies in the post marketing setting.
Identify similarities and/or differences between Member Companies in the use or development of DHTs (e.g., is there different infrastructure in place for Companies with greater numbers of DHTs?)	Understand level of experience with DHTs and infrastructure that supports DHTs.
Gather quantitative and qualitative data on PV function, process for collection of data regarding AE management, and/or patient safety.	Gain insights on the impact of DHTs on PV and/or patient safety.
Collect data on how Companies are now preparing for inevitable increase in DHTs.	Validate that DHTs is an evolving area that Companies are actively preparing for expansion.
Gather quantitative and qualitative data on what Member Companies feel are challenges and opportunities with DHTs without identifying specific systems or vendors.	Identify challenges and opportunities with DHTs to assist with prioritization of future solutions and/or topics for health authority interactions.

For this survey, DHTs include technologies, platforms, and systems that capture, store, or transmit health and/or clinical data in the post marketing setting (not in a clinical trial setting). DHTs support clinical decisions, patients with disease management or adherence to treatment, safety surveillance, risk management activities and/or approval activities. They can also be used to measure clinical outcomes, monitor patients, diagnostic purposes and/or therapeutic interventions to prevent, manage, or treat a medical disorder or disease. Examples include watches that can detect abnormal heart rate, apps that track disease symptoms (e.g., weight loss journey), mobile health platform that provides clinical data to physicians, telemedicine, artificial Intelligence in imaging analysis, etc. DHTs as intended in this survey do <u>not</u> include Company product specific webpages or social media intended for public awareness/education or promotional purposes. Although these digital platforms may collect health and/or clinical data, the primary intent of such sites is not for the purposes noted above for DHTs.

<u>This survey is not intended to collect information regarding the use of DHTs in the clinical trial/interventional study</u>.

Member Companies may opt out of responding to any questions that do not wish to answer for any reason (e.g., burdensome to obtain response, do not agree information is necessary, do not understand question and how to respond, companies feels that the question may ask for confidential or business sensitive information they are not comfortable disclosing etc.).

This survey is not intended to imply that any Member Company is not meeting current requirements. It is recognized that current regulatory requirements may be ambiguous or silent regarding specific requirements of DHTs. Therefore, it is expected that Member Companies may handle similar situations in different ways. Managing a situation different than choices available in questions (e.g., not having an appropriate choice) should not be interpreted as though a Member Company is managing a situation incorrectly.

The survey limits the use of free text field to decrease risk of identifying a Member Company and to better able to quantify data into a meaningful insight. Free text can be challenging to identify common themes and therefore, the Working Group has specifically designed the survey to not permit free text for key questions that are desirable to have quantifiable data. In a limited number of questions, free text is permissible when more information is desirable. Please ensure to not enter any information that may identify a Member Company or reveal proprietary information. A 3rd party project manager will be screening these responses prior to review of the data to ensure that it is not possible to identify a Member Company and/or that proprietary information was not shared. If there is identifiable/proprietary information, the response will be deleted or deidentified prior to evaluation of survey results.

In some responses you will be requested for a single response and in others you will be requested to select more than one option. If the directions do not indicate multiple answers, this is a single answer response.

To begin the survey on the next page, please select which Member Company you are representing for this survey. This information is only used for tracking purposes and is not included in the analysis of survey results. It will only be viewed by a 3rd party project manager who will be managing the distribution of the survey. This 3rd party will also perform an initial review of responses to ensure that Member Company identification is not revealed and/or proprietary information has not been shared and will aggregate responses, if necessary or appropriate.

Topic 8: PV Data from Digital Health Systems: Regulations, Implications and Opportunities

Member Company Survey

The purpose of this section is to collect information specific to the PV function (e.g., structure), patient safety and management of adverse event (AE) data.

This section should be responded to by applicable PV representatives.

This section is highly desirable to meet the Working Group objectives and it would be appreciated if this section is prioritized if time does not permit to complete the full survey.

1. Please select your Member Company. (Select one option)

- AbbVie
- Allergan
- Amgen
- Astellas
- AstraZeneca
- Bayer
- Boehringer Ingelheim
- Bristol-Myers Squibb
- EMD Serono/Merck KGaA
- GlaxoSmithKline
- Johnson & Johnson
- Eli Lilly

- MSD/Merck & Co.
- Novartis
- Novo Nordisk
- Pfizer
- Regeneron
- Roche
- Sanofi
- Shionogi
- UCB

2. How is PV function/department involved in the development of DHTs within your Company?

(Select one option)

- There is a PV role included in a dedicated DHT function (department who primary responsibility is to develop and launch new DHTs) within the Company
- PV is consulted to evaluate if the DHT has a PV impact as part of a dedicated DHT function's development process.
- PV is a member of a cross-functional working group that is formed with each DHT development.
- PV involvement in development is limited to DHTs who primary purpose is PV related (e.g., risk management activity required by health authority)
- PV is not involved in DHTs development.
- Decline to answer
- Other (Please specify) __________

3. Is there a requirement for a PV review (e.g., evaluate for AE reporting requirements, need for safety data monitoring plan, etc.) prior to launch of a DHT?

(Select one option)

- ◯ PV review is required for all DHTs prior to launch.
- ◯ PV Review is not required for DHTs; Company policies cover the requirements for the management of AE data with appropriate training.
- ◯ Depends on the type of DHTs.
- ◯ Depends on the objective (e.g., solution for risk management) of the DHT.
- ◯ Depends on whether AE data can be collected in the DHT (e.g., data is retrievable and clinical data could indicate that an AE occurred)
- ◯ Decline to answer

NOTE : Answer the below question only if answer to((Q#3 is **Depends on the type of DHTs.**))

4. If PV review is dependent on type of DHTs, what DHTs require PV review?

Select all that apply:

- ☐ Software as a medical device
- ☐ Connected Drug Delivery Devices
- ☐ Wearables
- ☐ Sensors
- ☐ Chatbot
- ☐ Virtual Reality/Augmented Reality tools
- ☐ Mobile apps
- ☐ Websites
- ☐ Decline to answer

☐ Other (Please specify) ____________

NOTE : Answer the below question only if answer to((Q#3 is **Depends on the objective (e.g., solution for risk management) of the DHT.**))

5. If PV review is dependent on the objective/primary purpose of the DHT, what is the objective of the DHT that requires PV review?

Select all that apply:

☐ The DHT supports a clinical decision.

☐ The DHT supports patients with disease management or adherence to treatment.

☐ The DHT is a tool used in safety surveillance.

☐ The DHT is used in risk management activities.

☐ The DHT measures clinical outcomes.

☐ The DHT monitors patients.

☐ The DHT is used for diagnostic purposes.

☐ The DHT is a therapeutic intervention to prevent, manage or treat a medical disorder or disease.

☐ Decline to answer

☐ Other (Please specify) ____________

6. Does your PV function have visibility to all DHTs that have been launched in the post marketing setting? (Select one option)

○ No, this information is not available to PV.

○ Yes, there is a Company-wide repository that can be accessed by multiple users regardless of function or location to identify all DHTs that have been launched in the Company.

- Yes, if needed, DHTs information can be made available to PV. Each function or location has its own method for tracking DHTs that they are managing and/or deployed.
- Decline to answer
- Other (Please specify) ___________

7. Does your Company document a specific plan for how AE data will be collected and reported for each DHT? (Select one option)

- Yes, a documented plan for the collection and reporting of AE data is done for each DHT.
- It depends on the specific DHTs.
- It depends on the objective of the DHT.
- No, there is a specific procedure and/or policy for management of AE data generated from DHTs.
- No, there are no specialized/specific instructions/policies for AE data generated from DHTs. Standard practices for management of AE data are followed.
- Decline to answer

8. For DHTs that collect disease symptoms/outcomes (e.g., laboratory values, weight gain/loss,.) that are entered by the patient, how does your Company determine if the data could be AE?

(Select one option)

- All abnormal values/symptoms are considered AE.
- There is a follow-up process to confirm if an AE occurred (e.g., patient receives a phone call).
- Instructions are provided to patients to report AEs via established spontaneous channels (e.g., phone #, website link), etc..). It is not assumed that entry of an abnormal value/symptom is an AE if patient has not reported this information as an AE.
- DHT has built in functionality for patients to report AEs. It is not assumed that an abnormal value/symptom is an AE if patient has not reported this information as an AE.
- Decline to answer
- Other (Please specify) ___________

9. For wearables, sensors and other DHTs that collect clinical data without a dependency on data entry (e.g., heart rate), how does your Company determine if data could be an AE?

(Select one option)

- ◯ All abnormal values/symptoms are considered AE.
- ◯ There is a follow-up process to confirm if an AE occurred (e.g., patient receives a phone call).
- ◯ Instructions are provided to patients to report AEs via established spontaneous channels (e.g., phone #, website link), etc..). It is not assumed that entry of an abnormal value/symptom is an AE if patient has not reported this information as an AE.
- ◯ DHT has built in functionality for patients to report AEs. It is not assumed that an abnormal value/symptom is an AE if patient has not reported this information as an AE.
- ◯ Decline to answer
- ◯ Other (Please specify) __________

10. In what DHT situations has your Company set-up mechanisms within the DHT (e.g., link to enter AE report, section to report AE, etc..) to report AEs directly to the Company?

(Select one option)

- ◯ Not applicable. No experience with this at this time.
- ◯ For DHTs that collect clinical data without dependency of data entry such as a wearable or sensor.
- ◯ All DHTs have built in functionality to report AEs. It is the Company standard.
- ◯ Varies depending on design and/or purpose of DHT.
- ◯ Decline to answer
- ◯ Other (Please specify) __________

11. Has your Company included automated alerts into DHTs to alert the Company of the potential of an adverse event?

For example, if the DHT detected an abnormal vital sign or laboratory value or increased frequency of a symptom, there is a notification to a Designee that there is a potential adverse event.

(Select one option)

- ○ Yes, alerts are set up with processes to reach out to the patient to verify if an adverse event has occurred.
- ○ Yes, alerts are set up to Designees to capture abnormal clinical data as adverse events and then standard follow-up process is followed.
- ○ No, alerts are not set up. Processes are in place to review data at regular intervals.
- ○ No, alerts are not set up as abnormal clinical data is not assumed to be an AE.
- ○ Decline to answer
- ○ Other (Please specify) __________

12. **Has you Company set up a process for additional aggregate safety reviews of data coming from a DHT (beyond routine signal detection activities)?**

    For example, on an annual basis, data coming from a single DHT is reviewed to determine if there are any safety concerns.

    Select all that apply:

- ☐ No, standard process for signal detection is followed.
- ☐ Yes, our Company has set up an aggregate safety data review process for a DHT.
- ☐ Depends on the purpose of the DHT (e.g., risk management activity)
- ☐ Depends on the type of DHT (e.g., wearable)
- ☐ Decline to answer
- ☐ Other (Please specify) ______________

NOTE : Answer the below question only if answer to((Q#12 is **Yes, our Company has set up an aggregate safety data review process for a DHT. OR Depends on the purpose of the DHT (e.g., risk management activity) OR Depends on the type of DHT (e.g., wearable)**))

13. Why was the decision made to set up a specific aggregate review of safety data from a DHT?

Select all that apply:

- ☐ Health authority request
- ☐ Risk management activity
- ☐ Signal Management
- ☐ Non-Interventional Study Analysis activity
- ☐ Decline to answer
- ☐ Other (Please specify) ____________

14. Does your Company have a selection within the safety database to identify that the source of the AE report is from a DHT?

(Select one option)

- ○ Yes
- ○ No
- ○ Decline to answer

NOTE : Answer the below question only if answer to((Q#14 is **Yes**))

15. If there is the ability to identify the source of an AE report in the database as a DHT, how is this achieved? (Select one option)

- ○ The process is similar to study set-up in the database where specific information about the DHT is collected to add to the database for future searches/selection.
- ○ There is a mechanism in the safety database to indicate this report is from DHT, but specific information is not collected (e.g., checkbox, drop down box, free text field with standard text/convention for entry)

◯ Decline to answer

◯ Other (Please specify) __________

NOTE : Answer the below question only if answer to((Q#14 is **Yes**))

16. What is the approximate volume of ICSRs generated from DHTs annually? (Select one option)

◯ Less than 1% of total volume of ICSRs annually

◯ Between 2 and 5 % of total volume of ICSRs annually

◯ Between 6 and 10% of total volume of ICSRs annually

◯ Greater than 10% of total volume of ICSRs annually

◯ Decline to answer

17. For each of the 10 challenges listed below, please order from most challenging to least challenging, '1' being the most challenging and '10' being the least challenging:

Note: You can drag and drop these challenges in order by clicking on the three lateral lines an dragging. Each challenge must be assigned their own ranking.

Limited knowledge/experience with the technology (e.g., digital literacy) : ________

Ambiguous health authority requirements : ________

Data management (collection, monitoring, processing) : ________

Capability of patients/HCPS to adopt/use the DHTs. : ________

Understanding privacy limitations/required content of consents. : ________

Determining if DHTs classifies as software as medical device. : ________

Having clear instructions for intended user. : ________

Ability to quantify a measure of success. : ________

Clinical validation (e.g., best method) : ________

Determination of whether an AE occurred. : ________

18. Is it challenging to determine if clinical/health data received from a DHT should be considered an AE? (Select one option)

- ○ Yes
- ○ No
- ○ Decline to answer

**NOTE :** Answer the below question only if answer to((Q#18 is **Yes**))

19. Why does your Company find it challenging to determine if an AE occurred?

Select all that apply:

- ☐ Lack of regulatory guidelines
- ☐ Lack of clinical guideline
- ☐ Insufficient information to associate event with product use.
- ☐ Decline to answer
- ☐ Other (Please specify) ____________

20. In the list provided, please select what your Company may find challenging when evaluating if all 4 elements of a valid ICSR has been met.

Select all that apply:

- ☐ N/A. Not a challenge.
- ☐ If reporter is identifiable.
- ☐ If a patient exists.
- ☐ Whether an AE has occurred.
- ☐ What product the person was using at the time of the event.
- ☐ Whether the information is credible (e.g., User error? Data entry error?)
- ☐ Decline to answer

21. After receipt of an initial ICSR from a DHT, does your Company have difficulty verifying AE data and/or obtaining additional medically relevant information (i.e., following up with reporter)?

Select the answer on the most common scenario your Company experiences:

(Select one option)

- ○ Yes, typically there is no means to privately contact the reporter.
- ○ Yes, there is not typically consent to obtain additional information (i.e., reporter declines to be contacted)
- ○ No, there is typically information available to follow-up with the reporter.
- ○ No, typically all medically relevant information has been provided.
- ○ Unable to respond as there is not one common scenario.
- ○ Decline to answer

22. From the following list, please select what your Company considers a challenge with the management of AE data collected from DHTs.

Select all that apply:

- [ ] Decision making on whether the data collected from the DHTs indicates an AE occurred.
- [ ] Quality of the safety data
- [ ] Ability to retrieve the AE data from the DHTs due to technical limitations.
- [ ] Understanding privacy restrictions in retrieving AE data (e.g., is data protected by physician/patient confidentiality, adequate consent to retrieve)
- [ ] Unclear regulatory requirements
- [ ] Decline to answer
- [ ] Other (Please specify) ______________

23. Is your Company anticipating that the volume of AEs will increase with the use of DHTs? (Select one option)

○ Yes

○ No

○ Not sure

○ Decline to answer

Topic 8: PV Data from Digital Health Systems: Regulations, Implications and Opportunities

Member Company Survey

The purpose of this section is to collect information specific to PV quality and compliance.

This section should be responded to by applicable PV representatives.

This section is highly desirable to meet the Working Group objective and it would be appreciated if this section is prioritized if time does not permit to complete the full survey.

24. From a PV perspective, would you agree that a DHT could improve the quality of safety data received by the Company (e.g., the data collected enables benefit/risk decisions)?

(Select one option)

- ○ PV function believes that there is significant potential in using DHTs to collect quality safety data.
- ○ PV function believes there is some potential in using DHTs to collect quality safety data, but we have concerns that there will also be increased noise.
- ○ PV function does not see potential in using DHTs to collect quality safety data.
- ○ PV function has limited or no experience with DHTs to see potential of DHTs to collect quality safety data.
- ○ Not sure
- ○ Decline to answer

25. Does your Company have experience with health authority PV inspection queries related to DHTs (e.g., targeted DHT inspection, focused sessions during routine PV inspection, specific queries about DHTs, etc.)?

(Select one option)

- ○ Yes

- ○ No
- ○ Decline to answer

NOTE : Answer the below question only if answer to((Q#25 is **Yes**))

26. What were the PV health authority's area of interest during this inspection?

Select all that apply:

- ☐ Correct classification of the DHTs (e.g., correctly classified as a medical device)
- ☐ Management of safety data collected from DHTs in meeting ICSR requirements.
- ☐ Management of safety data collected in DHTs in performing signal detection activities.
- ☐ Management of safety data from DHTs in Regulatory Aggregate Reports (e.g., PSUR)
- ☐ Use of DHTs in Risk Management activities
- ☐ Use of DHTs in non-interventional Studies
- ☐ Use of DHTS in Post Authorization Safety Studies
- ☐ How Company triggers HCP interaction when a predefined alert has been met (e.g., abnormal clinical value that is considered significant)
- ☐ Decline to answer
- ☐ Other (Please specify) ____________

NOTE : Answer the below question only if answer to((Q#25 is **Yes**))

27. If applicable, what type of observations were cited specific to DHTs.

Select all that apply:

- [ ] Not applicable, no observations/concerns
- [ ] Incorrect classification of DHT
- [ ] Management of Safety Data
- [ ] Signal Detection
- [ ] Risk Management activities
- [ ] Non-interventional study related.
- [ ] Decline to answer

Topic 8: PV Data from Digital Health Systems: Regulations, Implications and Opportunities

Member Company Survey

The purpose of this section is to collect general information about where Member Companies focus their DHT development/use.

This section is not specific to PV and will require input from other functions such as technology support services and organizations who own the software development life cycle process in your Company or who primarily initiate the launch of DHTs (e.g., Marketing/Commercial). This section is requested to provide general background/context to support further publications and/or health authority interaction.

28. **Select what DHTs your Company has deployed/launched in the post marketing setting that are designed specifically to collect patient health and/or clinical data for the purpose of supporting clinical decisions, patients with disease management or adherence to treatment, safety surveillance, risk management activities or approval activities. The purpose may also be to measure clinical outcomes, monitor patients, diagnostic purposes and/or therapeutic interventions to prevent, manage or treat a medical disorder or disease.**

IMPORTANT:

This question is not intended to identify technologies that may collect health and/or clinical data because of open text fields, such as on a company product specific webpage or social media intended for general public awareness/education or promotional purposes.

NOTE: This list below is representative of common DHTs. It is not intended to be all inclusive.

Select all that apply:

- ☐ Software as a medical device
- ☐ Connected Drug Delivery Devices
- ☐ Wearables
- ☐ Sensors
- ☐ Chatbot

- [ ] Virtual Reality/Augmented Reality tools
- [ ] Mobile apps
- [ ] Websites
- [ ] Decline to answer
- [ ] None of the above

NOTE : Answer the below question only if answer to((Q#28 is **Software as a medical device**))

29. What is the approximate number of SOFTWARE AS A MEDICAL DEVICE that your Company has deployed in the post marketing setting from 01-AUG-2021 to 01-AUG-2023 (i.e., last 2 years)?

(Select one option)

- ○ Less than 10
- ○ Between 10-100
- ○ Between 101-500
- ○ Between 501-1000
- ○ Greater than 1000
- ○ Decline to answer

NOTE : Answer the below question only if answer to((Q#29 is answered))

30. What is the approximate number of years that SOFTWARE AS A MEDICAL DEVICE has been used in the post marketing setting?

(Select one option)

- ○ Less than 1 year

- 1 to 3 years
- 4 to 6 years
- 7 to 10 years
- Greater than 10 years
- Decline to answer

NOTE : Answer the below question only if answer to((Q#28 is **Connected Drug Delivery Devices**))

31. What is the approximate number of CONNECTED DRUG DELIVERY DEVICES that your Company has deployed in the post marketing setting from 01-AUG-2021 to 01-AUG-2023 (i.e., last 2 years)? (Select one option)

- Less than 10
- Between 10-100
- Between 101-500
- Between 501-1000
- Greater than 1000
- Decline to answer

NOTE : Answer the below question only if answer to((Q#31 is answered))

32. What is the approximate number of years that CONNECTED DRUG DELIVERY DEVICES have been used in the post marketing setting? (Select one option)

- Less than 1 year
- 1 to 3 years

- 4 to 6 years
- 7 to 10 years
- Greater than 10 years
- Decline to answer

NOTE : Answer the below question only if answer to((Q#28 is **Wearables**))

33. What is the approximate number of WEARABLES that your Company has deployed in the post marketing setting from 01-AUG-2021 to 01-AUG-2023 (i.e., last 2 years)? (Select one option)

- Less than 10
- Between 10-100
- Between 101-500
- Between 501-1000
- Greater than 1000
- Decline to answer

NOTE : Answer the below question only if answer to((Q#33 is answered))

34. What is the approximate number of years that WEARABLES have been used in the post marketing setting? (Select one option)

- Less than 1 year
- 1 to 3 years
- 4 to 6 years

- 7 to 10 years
- Greater than 10 years
- Decline to answer

NOTE : Answer the below question only if answer to((Q#28 is **Sensors**))

35. What is the approximate number of SENSORS that your Company has deployed in the post marketing setting from 01-AUG-2021 to 01-AUG-2023 (i.e., last 2 years)? (Select one option)

- Less than 10
- Between 10-100
- Between 101-500
- Between 501-1000
- Greater than 1000
- Decline to answer

NOTE : Answer the below question only if answer to((Q#35 is answered))

36. What is the approximate number of years that SENSORS have been used in the post marketing setting? (Select one option)

- Less than 1 year
- 1 to 3 years
- 4 to 6 years
- 7 to 10 years

- Greater than 10 years
- Decline to answer

NOTE : Answer the below question only if answer to((Q#28 is **Chatbot**))

37. What is the approximate number of CHATBOTS that your Company has deployed in the post marketing setting from 01-AUG-2021 to 01-AUG-2023 (i.e., last 2 years)? (Select one option)

- Less than 10
- Between 10-100
- Between 101-500
- Between 501-1000
- Greater than 1000
- Decline to answer

NOTE : Answer the below question only if answer to((Q#37 is answered))

38. What is the approximate number of years that CHATBOTS have been used in the post marketing setting? (Select one option)

- Less than 1 year
- 1 to 3 years
- 4 to 6 years
- 7 to 10 years
- Greater than 10 years

- ○ Decline to answer

NOTE : Answer the below question only if answer to((Q#28 is **Virtual Reality/Augmented Reality tools**))

39. What is the approximate number of VIRTUAL REALITY / AUGMENTED REALITY TOOLS that your Company has deployed in the post marketing setting from 01-AUG-2021 to 01-AUG-2023 (i.e., last 2 years)? (Select one option)

- ○ Less than 10
- ○ Between 10-100
- ○ Between 101-500
- ○ Between 501-1000
- ○ Greater than 1000
- ○ Decline to answer

NOTE : Answer the below question only if answer to((Q#39 is answered))

40. What is the approximate number of years that VIRTUAL REALITY / AUGMENTED REALITY TOOLS have been used in the post marketing setting? (Select one option)

- ○ Less than 1 year
- ○ 1 to 3 years
- ○ 4 to 6 years
- ○ 7 to 10 years
- ○ Greater than 10 years
- ○ Decline to answer

NOTE : Answer the below question only if answer to((Q#28 is **Mobile apps**))

41. What is the approximate number of MOBILE APPS that your Company has deployed in the post marketing setting from 01-AUG-2021 to 01-AUG-2023 (i.e., last 2 years)? (Select one option)

- ○ Less than 10
- ○ Between 10-100
- ○ Between 101-500
- ○ Between 501-1000
- ○ Greater than 1000
- ○ Decline to answer

NOTE : Answer the below question only if answer to((Q#41 is answered))

42. What is the approximate number of years that MOBILE APPS have been used in the post marketing setting? (Select one option)

- ○ Less than 1 year
- ○ 1 to 3 years
- ○ 4 to 6 years
- ○ 7 to 10 years
- ○ Greater than 10 years
- ○ Decline to answer

NOTE : Answer the below question only if answer to((Q#28 is **Websites**))

43. What is the approximate number of WEBSITES that your Company has deployed in the post marketing setting from 01-AUG-2021 to 01-AUG-2023 (i.e., last 2 years)? (Select one option)

- ○ Less than 10
- ○ Between 10-100
- ○ Between 101-500
- ○ Between 501-1000
- ○ Greater than 1000
- ○ Decline to answer

NOTE : Answer the below question only if answer to((Q#43 is answered))

44. What is the approximate number of years that WEBSITES have been used in the post marketing setting? (Select one option)

- ○ Less than 1 year
- ○ 1 to 3 years
- ○ 4 to 6 years
- ○ 7 to 10 years
- ○ Greater than 10 years
- ○ Decline to answer

45. What therapeutic areas/disease states does your Company deploy DHTs?

Select all that apply:

- [ ] Autoimmune
- [ ] Cardiovascular
- [ ] Endocrine
- [ ] Gastrointestinal
- [ ] Infectious
- [ ] Mental Health
- [ ] Neurology
- [ ] Oncology/Hematology
- [ ] Rare Disease
- [ ] Respiratory
- [ ] Other
- [ ] Decline to answer

46. Does your Company deploy DHTs for acute illnesses and/or diseases if they state that duration is less than 1 year (e.g., certain cancers) in the post marketing setting (not as part of a clinical trial)?

(Select one option)

○ Yes

○ No

○ Decline to answer

47. For what purpose was a DHT(s) deployed/launched in your Company in the post marketing setting?

Select all that apply:

- [ ] Support clinical decisions.
- [ ] Support patients with disease management or adherence to treatment
- [ ] Safety surveillance
- [ ] Risk management activities
- [ ] Measure clinical outcomes.
- [ ] Monitor patients.
- [ ] Diagnostic purposes
- [ ] Therapeutic interventions to prevent, manage or treat a medical disorder or disease.
- [ ] Decline to answer
- [ ] Other (Please specify) ____________

48. Has your Company measured whether DHTs improved patient adherence and/or safety outcomes?

This response is not intended to include DHTs used in clinicial trials.

(Select one option)

- ○ Yes
- ○ No
- ○ Not sure
- ○ Not applicable
- ○ Decline to answer

NOTE : Answer the below question only if answer to((Q#48 is **Yes**))

49. **How was improved patient adherence and/or safety outcomes measured?**

Select all that apply:

- [ ] Internally developed methodology
- [ ] Patient Reported Outcome Measure
- [ ] Digital biomarker/endpoint
- [ ] Measure against an external standard (e.g., medical best practice, approved clinical measure)
- [ ] Decline to answer

50. **Does your Company feel that DHTs are beneficial in the post market setting?**

(Select one option)

- ○ No benefit
- ○ Low benefit
- ○ Moderate benefit
- ○ High benefit
- ○ Unsure
- ○ Decline to answer

NOTE : Answer the below question only if answer to((Q#50 is **Low benefit OR Moderate benefit OR High benefit**))

51. **What benefit has your Company seen by deploying DHTs?**

(Select one option)

- Improved patient adherence/outcomes
- Enhanced safety surveillance and/or signal detection of adverse events
- Improved public health awareness.
- Improved health equity
- Decline to answer
- Other (Please specify) __________

52. Has your Company combined patient clinical/health data from DHTs with electronic health records for analysis and/or clinical decision purposes in the post marketing setting? (Select one option)

- Yes
- No, difficulties with interoperability (ability to connect data sources)
- No privacy constraints.
- No, business decision not to pursue.
- Decline to answer
- No, Other (Please specify) __________

Topic 8: PV Data from Digital Health Systems: Regulations, Implications and Opportunities

Member Company Survey

The purpose of this section is to collect general information about developing and implementing DHTs level of experience with DHTs, and infrastructure to support DHTs in the post marketing setting.

This section is not specific to PV and may require input from other functions such as technology support services and organizations who own the software development life cycle process in your Company or who primarily initiate the launch of DHTs (e.g., Marketing/Commercial). This section is requested to provide general background/context to support further publications and/or health authority interaction.

53. What organization in your Company initiates (e.g., project lead, owning organization) the development and deployment of DHTs in the post marketing setting?

Select all that apply:

- ☐ Commercial/Marketing
- ☐ Medical Affairs
- ☐ Research & Development
- ☐ Digital Health/Innovation
- ☐ PV
- ☐ Other
- ☐ Decline to answer

54. Do you have a quality management system or process that defines how you develop a DHT for post marketing setting? (Select one option)

- ◯ Yes, we have a process / quality management system for development of a DHT.
- ◯ No, we do not have a process / quality management system specific for development of DHT (e.g., general process for development of digital assets, regardless of purpose)
- ◯ Decline to answer
- ◯ Other (Please specify) __________

55. During the development of a DHT, what validation do you perform? (Select one option)

- ◯ Technical Validation
- ◯ Clinical Validation
- ◯ Both technical and clinical validation
- ◯ Unsure
- ◯ Decline to answer

56. Who develops DHTs in the post marketing setting in your Company?

Select all that apply:

- ☐ In-house
- ☐ Collaboration between in house and outside vendor
- ☐ DHTs are not developed by the Company.
- ☐ Decline to answer

57. Does your Company have a dedicated cross-functional group to review DHT concepts (before development begins) in a post marketing setting? (Select one option)

- ◯ Yes
- ◯ No
- ◯ Decline to answer

**NOTE :** Answer the below question only if answer to((Q#57 is **Yes**))

58. Please select all the functions that are part of the concept review:

Select all that apply:

- ☐ Project Management
- ☐ IT
- ☐ Quality (manufacturing, group who receives product complaints)
- ☐ Medical Affairs
- ☐ Commercial/Marketing
- ☐ Pharmacovigilance (PV)
- ☐ Regulatory Affairs
- ☐ Digital Health/Innovation
- ☐ Legal
- ☐ Decline to answer
- ☐ Other (Please specify) ______________

59. What functional approvals are required prior to launch of a DHTs in a post marketing setting?

Select all that apply:

- ☐ Project Management
- ☐ IT
- ☐ Quality/Manufacturing
- ☐ Medical Affairs
- ☐ Commercial/Marketing
- ☐ Local PV
- ☐ Global PV
- ☐ Regulatory Affairs
- ☐ Digital Health/Innovation
- ☐ Legal
- ☐ Decline to answer
- ☐ Other (Please specify) ____________

60. Is there a centralized repository that tracks what DHTs have been developed and launched? (Select one option)

- ○ Yes
- ○ No
- ○ Decline to answer

NOTE : Answer the below question only if answer to((Q#60 is **Yes**))

61. If there is a centralized repository, what type of information is tracked in the centralized repository?

Select all that apply:

- ☐ Type of DHTs (wearable, sensor, app, etc.
- ☐ Regulatory Classification (e.g., whether medical device)
- ☐ Purpose
- ☐ Status (e.g., in development, active, decommissioned)
- ☐ Relevant dates to Company (e.g., launch date, decommission date)
- ☐ Number of Users
- ☐ If data is retrievable
- ☐ Decline to answer
- ☐ Other (Please specify) ______________

62. What types of partnerships or collaborations is your Company pursuing in order to advance the research, development and/or enhancement of DHTs?

Select all that apply:

- ☐ Our Company is partnering with technology companies to develop and implement DHTs.
- ☐ Our Company is collaborating with academic institutions to advance research and development in DHTs.
- ☐ Our Company is partnering with healthcare providers to pilot and implement DHTs in clinical settings.
- ☐ Our Company is collaborating with patient advocacy groups to ensure patient-centered design and development of DHTs.

- [ ] Our Company is partnering with regulatory agencies to ensure compliance and adherence to regulations for DHTs.

- [ ] Our Company is pursuing partnerships with other organizations in the healthcare industry to collectively advance DHTs.

- [ ] Our Company has no active partnership.

- [ ] Decline to answer

63. How is your organization engaging with patients and healthcare providers to ensure successful adoption and use of DHTs?

Select all that apply:

- [ ] Our Company conducts surveys and focus groups with patients and healthcare providers to understand their needs and preferences for DHTs.

- [ ] Our Company involves patients and healthcare providers in the design and development of DHTs to ensure they are user-friendly and meet their needs.

- [ ] Our Company offers training and education to patients and healthcare providers to ensure they are comfortable and competent in using digital health technologies.

- [ ] Our Company provides ongoing support and resources to patients and healthcare providers to ensure successful adoption and use of DHTs.

- [ ] Our Company establishes partnerships with patient advocacy groups and healthcare organizations to promote awareness and adoption of DHTs.

- [ ] Our Company has a dedicated patient engagement team that focuses on ensuring successful adoption and use of DHTs.

- [ ] Our Company leverages social media and other digital platforms to engage with patients and healthcare providers and promote adoption of DHTs.

- [ ] Not sure

- [ ] Decline to answer

Topic 8: PV Data from Digital Health Systems: Regulations, Implications and Opportunities

Member Company Survey

The purpose of this section is to collect information to understand how Member Companies are preparing for expansion of DHTs and to identify potential opportunities with DHTs that collect patient health/clinical data in the post marketing setting.

This section is intended for the PV function to respond to gain PV perspective on future opportunities.

64. Is there an opportunity to improve the process of launching DHTs within your Company?

(Select one option)

- ○ There are significant opportunities to improve the process to launch DHTs.
- ○ There are some opportunities to improve the process to launch DHTs.
- ○ Our process to launch DHTs is adequate, and our Company does not see many opportunities for improvement.
- ○ N/A Our Company does not have experience with DHTs.
- ○ Decline to answer
- ○ Other (Please specify) __________

65. Is your Company restructuring in PV to specifically support DHTs? (Select one option)

- ○ Our Company has no changes or planned future changes.
- ○ Our Company is currently evaluating whether changes are required to better support DHTs.
- ○ Our Company has created new roles and/or restructuring is occurring to support DHTs.
- ○ Our Company will be decreasing resources/focus in DHTs.
- ○ Not sure

- ◯ Decline to answer

66. Is your Company providing education to PV personnel on DHTs?

(Select one option)

- ◯ Our Company provides comprehensive education to PV personnel on DHTs.
- ◯ Our Company provides some education to PV personnel on DHTs, but it could be more comprehensive.
- ◯ Our Company has not provided education to PV personnel on DHTs, but we are considering it.
- ◯ Our Company does not provide education to PV personnel on DHTs.
- ◯ Not Sure
- ◯ Decline to answer

67. Does your Company have a digital transformation roadmap to encourage expansion/adoption of DHTs that includes PV engagement/involvement?

(Select one option)

- ◯ Our Company has a clear digital transformation roadmap that includes PV engagement/involvement.
- ◯ Our Company has a digital transformation roadmap, but it does not currently include PV engagement/involvement.
- ◯ Our Company is in the process of developing a digital transformation roadmap that will include PV engagement/involvement.
- ◯ Our Company does not have a digital transformation roadmap.
- ◯ Not Sure
- ◯ Decline to answer

68. Do you have a PV specific strategic plan/framework to support DHTs and/or a specific roadmap for PV to prepare for management of safety data from digital technologies? (Select one option)

- ◯ Our Company has a PV specific strategic plan/framework in place to support DHTs, including a roadmap for managing safety data from digital technologies.

- Our Company has a PV specific strategic plan/framework in place, but it does not currently include a roadmap for managing safety data from digital technologies.
- Our Company is in the process of developing a PV specific strategic plan/framework to support DHTs, including a roadmap for managing safety data from digital technologies.
- Our Company does not have a PV specific strategic plan/framework in place to support DHTs, nor do we have a roadmap for managing safety data from digital technologies.
- Not Sure
- Decline to answer

69. Do you feel that your Company is well positioned to manage the increased use of DHTs and the respective increased volume of safety reports? (Select one option)

- Our Company is well positioned to manage the increased use of DHTs and the respective increased volume of safety reports.
- Our Company has some concerns about our ability to manage the increased use of DHTs and the respective increased volume of safety reports.
- Our Company does not feel well positioned to manage the increased use of DHTs and the respective increased volume of safety reports.
- Our Company is not currently using DHTs in our PV process, so this question is not applicable.
- Not Sure
- Decline to answer

70. Would your Company support the need for a consolidated glossary to define DHT terminology? (Select one option)

- Yes
- No
- Decline to answer

NOTE : Answer the below question only if answer to((Q#70 is **No**))

71. Since you answered no, please explain why.

72. Would your Company support the need for a reference library of health authority regulations and/or guidance specific to DHTs? (Select one option)

- ○ Yes
- ○ No
- ○ Decline to answer

NOTE : Answer the below question only if answer to((Q#72 is **No**))

73. Since you answered no, please explain why.

---

74. Would your Company support the need for a solution providing general principles/points to consider regarding privacy for implementation of DHTs? (Select one option)

- ○ Yes
- ○ No
- ○ Decline to answer

NOTE : Answer the below question only if answer to((Q#74 is **No**))

75. Since you answered no, please explain why.

---

76. Would your Company support the need for a solution that provides general principles/points to consider on how to determine if clinical/health data should be considered an AE? (Select one option)

- ◯ Yes
- ◯ No
- ◯ Decline to answer

NOTE : Answer the below question only if answer to((Q#76 is **No**))

77. Since you answered no, please explain why.

---

78. Does your Company have any recommendations on what type of tool/solution would assist with the management of patient clinical/health data collected from DHTs?
